# Facial discoid dermatosis successfully treated with topical ruxolitinib: A case report

**DOI:** 10.1016/j.jdcr.2026.07.039

**Published:** 2026-07-30

**Authors:** Aline Garcia, Julie Archimbaud, Jean Kanitakis, Axel Patrice Villani, Denis Jullien, Cécile Lesort

**Affiliations:** a Department of Dermatology, Edouard Herriot Hospital, Hospices Civils de Lyon, Lyon, France; b Claude Bernard - Lyon 1 University, Lyon, France; c Department of Pathology, Lyon-Sud Hospital, Hospices Civils de Lyon, Pierre-Bénite, France; d CIRI, Centre International de Recherche en Infectiologie (Team Epidermal Immunity and Allergy), INSERM, U1111, Univ Lyon, Université de Lyon 1, École Normale Supérieure de Lyon, CNRS, UMR 5308, Lyon, France

**Keywords:** facial discoid dermatosis, JAK inhibitor, pityriasis rubra pilaris, ruxolitinib, topical treatment

## Introduction

Facial discoid dermatosis (FDD) is a rare dermatosis first described in 2010 by Ko et al.^1^ It is characterized by well-demarcated, annular, pink-orange, scaly papules and plaques localized on the face and occasionally the neck. FDD remains a diagnostic and therapeutic challenge, as it is often misdiagnosed as psoriasis, seborrheic dermatitis, discoid lupus erythematosus, pityriasis rubra pilaris (PRP), or granulomatous rosacea, and is frequently resistant to conventional therapies.^2^ Ruxolitinib is a topical selective JAK1/2 inhibitor approved for non-segmental vitiligo,^3^ and has also demonstrated efficacy in atopic dermatitis.^4^ Herein, we report, to the best of our knowledge, the first case of FDD successfully treated with topical ruxolitinib, based on a comprehensive PubMed search from January 2010 to March 2026.

## Case report

A 60-year-old man presented with a 3-month history of asymptomatic facial lesions. Physical examination revealed multiple well-demarcated, round, erythematous-orange papules and plaques involving the face and neck (Fig 1, *A*). No palmoplantar keratoderma, islands of spared skin, or follicular papules on the dorsa of the fingers were observed. The patient had no significant medical history. Previous treatments, including topical corticosteroids (betamethasone cream) for 1 month, topical ivermectin for 2 months, doxycycline 200 mg/d for 3 months, and oral ivermectin, had been ineffective. Differential diagnoses included discoid lupus erythematosus, tumid lupus erythematosus, psoriasis, seborrheic dermatitis, follicular mucinosis, and FDD. Histological examination of a skin biopsy showed psoriasiform acanthosis with focal parakeratosis and follicular plugging, along with a moderate superficial perivascular lymphocytic infiltrate (Fig 2). Notably, neither *Demodex folliculorum* nor granulomas were found. Alcian blue staining, performed to rule out follicular mucinosis, was negative. Direct immunofluorescence was negative. Autoimmune laboratory testing, including antinuclear antibodies, was unremarkable. Following multidisciplinary discussion, a diagnosis of FDD was established. Given the absence of validated conventional therapy, the failure of multiple topical agents, and the rather limited extent of skin involvement, off-label ruxolitinib 1.5% cream was initiated twice daily (thin layer, approximately 10 g/week) after informed consent. Complete clearance was observed within 3 weeks of treatment initiation (Fig 1, *B*). No adverse events were reported. The dosing was subsequently tapered to once-daily ruxolitinib cream application, which resulted in sustained remission. No recurrence was observed at the 4-month follow-up.

**Fig 1:**
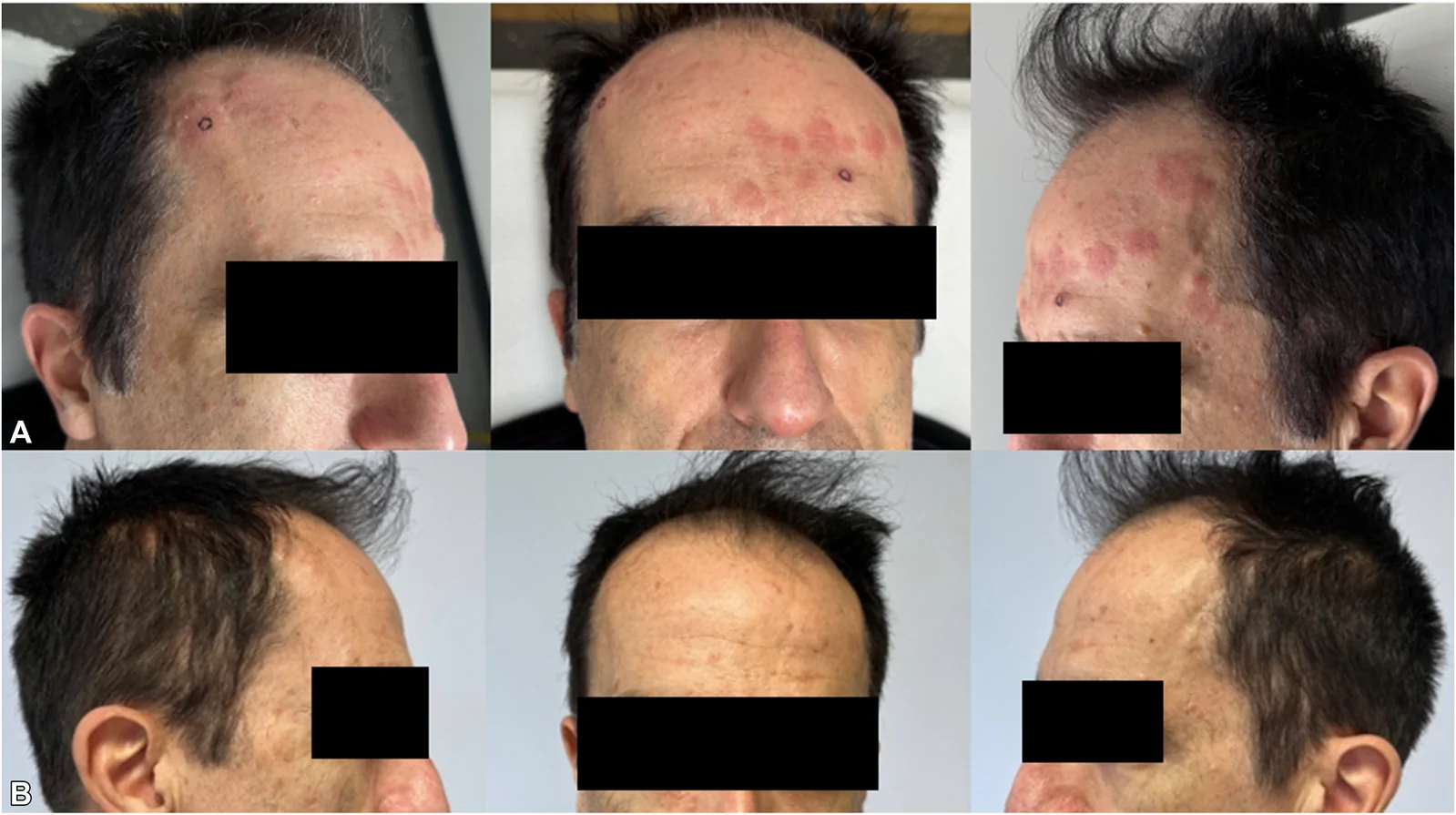
**A,** Clinical appearance of FDD before treatment: Multiple well-demarcated, round, erythematous-orange, slightly scaly papules and plaques on the face and neck. **B,** Clinical appearance 3 weeks after treatment initiation: complete remission of FDD was achieved with ruxolitinib 1.5% cream applied twice daily, without any concomitant treatment.

**Fig 2:**
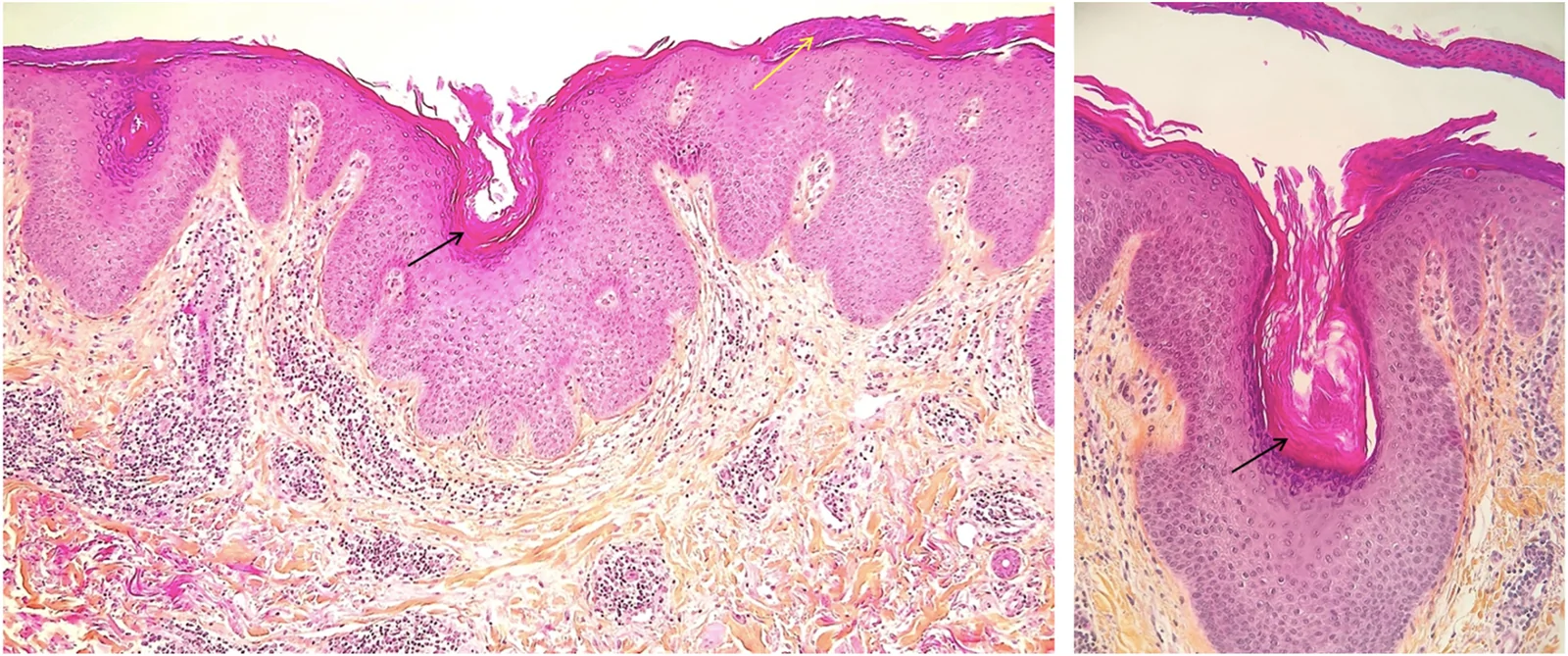
Histopathologic examination of a skin biopsy shows psoriasiform acanthosis with focal parakeratosis (*yellow arrow*) and follicular plugging (*black arrows*), along with a moderately-dense superficial perivascular lymphocytic infiltrate (hematoxylin-eosin-saffron staining, 5-μm section, original magnification [×100]).

## Discussion

FDD is a recently described facial dermatosis with an unclear pathogenesis. A familial case was recently described, suggesting a possible contribution of genetic factors.^5^ The largest case series to date included 13 patients, predominantly women, presenting with chronic, treatment-resistant facial papulosquamous lesions.^6^ A comprehensive review identified 27 reported cases, but no consistent effective therapy.^2^ Histopathologic findings in FDD include acanthosis, parakeratosis, follicular plugging and a moderate superficial lymphocytic infiltrate, ie features overlapping with psoriasis, seborrheic dermatitis and pityriasis rubra pilaris.^7^ Based on these similarities, some authors have proposed that FDD may represent a localized facial variant of PRP. Notably, progression from FDD to classic PRP has been reported in 1 case, supporting a potential pathogenic continuum.^7^ From a pathophysiologic perspective, the inflammatory infiltrate observed in FDD suggests a T-cell-mediated process. Although the cytokinic profile of FDD has not been specifically characterized, its clinicopathologic overlap with psoriasis and PRP raises the possibility of involvement of Th1/Th17-driven pathways. While IL-17A signals through the Act1/TRAF6/NF-κB pathway rather than JAK/STAT, several Th1/Th17-related cytokines, including IFN-γ, IL-22, and IL-23, do signal through the JAK/STAT pathway. This provides a biologic rationale for the use of JAK inhibitors in this condition.

The treatment of FDD remains challenging. Topical treatments—including corticosteroids, calcineurin inhibitors, antifungal agents, and topical sirolimus—have shown limited efficacy.^2^^,^^6^ Topical calcipotriol/betamethasone, however, has provided some benefit in several reported cases.^8^

Among systemic treatments, acitretin has provided benefit in isolated cases.^7^ Oral isotretinoin has recently been reported to achieve complete response in 1 patient.^9^ Biologic therapies have also been explored: ustekinumab, an IL-12/23 inhibitor, led to improvement in 1 case,^10^ while secukinumab, an IL-17A inhibitor, was recently reported to improve treatment-resistant FDD.^11^ However, these systemic approaches may not be ideal for a disease limited to the face, particularly in older patients or those with comorbidities. The rapid and complete response observed with topical ruxolitinib in our patient is notable, given the typically chronic and refractory course of FDD. The improvement achieved within 3 weeks suggests a direct anti-inflammatory effect, rather than a delayed keratinization-modulating mechanism, as seen with retinoids. In addition, the facial localization of FDD makes topical JAK inhibition particularly suitable, allowing targeted treatment while minimizing systemic exposure. Topical ruxolitinib was well tolerated in our patient, with no adverse events, namely no folliculitis or acne,^3^ despite active surveillance for these effects. Sustained remission after tapering to once-daily application further supports the potential utility of ruxolitinib as a maintenance therapy. This report has limitations. Firstly, it describes a single case with limited follow-up duration. Secondly, spontaneous remission cannot be entirely excluded, although the rapid response following multiple treatment failures makes this unlikely. Thirdly, no immunohistochemical or molecular studies were performed to confirm activation of the JAK/STAT pathway. Despite these limitations, this case suggests that topical JAK inhibition may represent a promising therapeutic option for FDD. Further studies are needed to better characterize the pathogenesis of FDD and to evaluate the efficacy and safety of JAK inhibitors in larger cohorts.

### Declaration of generative AI and AI-assisted technologies in the writing process

None.

## Conflicts of interest

None disclosed.
